# Beyond words: From jaguar population trends to conservation and public policy in Mexico

**DOI:** 10.1371/journal.pone.0255555

**Published:** 2021-10-06

**Authors:** Gerardo Ceballos, Heliot Zarza, José F. González-Maya, J. Antonio de la Torre, Andrés Arias-Alzate, Carlos Alcerreca, Horacio V. Barcenas, Gerardo Carreón-Arroyo, Cuauhtémoc Chávez, Carlos Cruz, Daniela Medellín, Andres García, Marco Antonio-García, Marco A. Lazcano-Barrero, Rodrigo A. Medellín, Oscar Moctezuma-Orozco, Fernando Ruiz, Yamel Rubio, Victor H. Luja, Erik Joaquín Torres-Romero

**Affiliations:** 1 Laboratorio de Ecología y Conservación de Fauna Silvestre, Instituto de Ecología, Universidad Nacional Autónoma de México, Ciudad Universitaria, Coyoacán, Ciudad de México, México; 2 Departamento de Ciencias Ambientales, Universidad Autónoma Metropolitana, Unidad Lerma, CBS, Lerma de Villada, México; 3 Proyecto de Conservación de Aguas y Tierras, ProCAT Colombia/Costa Rica, Bogotá, Colombia; 4 Laboratorio de Ecología y Conservación de Vertebrados Terrestres, Instituto de Ecología, Universidad Nacional Autónoma de México, Ciudad Universitaria, Ciudad de México, México; 5 Bioconciencia A.C., Ciudad de México, México; 6 Universidad CES, Facultad de Ciencias y Biotecnología, Medellín, Antioquia, Colombia; 7 Biocenosis, Mérida, Yucatán, México; 8 Facultad de Ciencias, Universidad Nacional Autónoma de México, Ciudad Universitaria, Ciudad de México, México; 9 Naturalia A.C., Hermosillo, Sonora, México; 10 Universidad de Alicante, Campus San Vicente del Raspeig, Alicante, España; 11 Estación de Biología Chamela, Instituto de Biología, Universidad Nacional Autónoma de México, San Patricio-Melaque, La Huerta, Jalisco, México; 12 Facultad de Ciencias Políticas y Sociales, Universidad Autónoma de Querétaro, Querétaro, México; 13 Reserva Ecológica El Edén A.C., Cancún, Quintana Roo, México; 14 Naturalia A.C., Col. Ampliación Nápoles, Benito Juárez, Ciudad de México, México; 15 Centro de Investigaciones Biológicas, Instituto de Ciencias Básicas e Ingeniería, Universidad Autónoma del Estado de Hidalgo, Ciudad del Conocimiento, Col. Carboneras, Mineral de la Reforma, Hidalgo, México; 16 Facultad de Biología, Universidad Autónoma de Sinaloa, Ciudad Universitaria, Culiacán, Sinaloa, México; 17 Unidad Academica de Turismo, Universidad Autonoma de Nayarit, Ciudad de la Cultura, Tepic, Nayarit; Indiana State University, UNITED STATES

## Abstract

The jaguar (*Panthera onca*) is one of the most threatened carnivores in the Americas. Despite a long history of research on this charismatic species, to date there have been few systematic efforts to assess its population size and status in most countries across its distribution range. We present here the results of the two National Jaguar Surveys for Mexico, the first national censuses in any country within the species distribution. We estimated jaguar densities from field data collected at 13 localities in 2008–2010 (2010 hereafter) and 11 localities in 2016–2018 (2018 hereafter). We used the 2010 census results as the basis to develop a National Jaguar Conservation Strategy that identified critical issues for jaguar conservation in Mexico. We worked with the Mexican government to implement the conservation strategy and then evaluated its effectivity. To compare the 2010 and 2018 results, we estimated the amount of jaguar-suitable habitat in the entire country based on an ecological niche model for both periods. Suitable jaguar habitat covered ~267,063 km^2^ (13.9% of the country’s territory) in 2010 and ~ 288,890 km^2^ (~14.8% of the country’s territory) in 2018. Using the most conservative density values for each priority region, we estimated jaguar densities for both the high and low suitable habitats. The total jaguar population was estimated in ~4,000 individuals for 2010 census and ~4,800 for the 2018 census. The Yucatan Peninsula was the region with the largest population, around 2000 jaguars, in both censuses. Our promising results indicate that the actions we proposed in the National Jaguar Conservation Strategy, some of which have been implemented working together with the Federal Government, other NGO’s, and land owners, are improving jaguar conservation in Mexico. The continuation of surveys and monitoring programs of the jaguar populations in Mexico will provide accurate information to design and implement effective, science-based conservation measures to try to ensure that robust jaguar populations remain a permanent fixture of Mexico’s natural heritage.

## Introduction

The loss of biological diversity is one of the most severely threatening global environmental problems caused by human activities. Thousands of species and millions of populations become extinct every year [1,2]. Populations of large mammals, birds, and other vertebrates have been decimated in the last decades, and it is estimated that more than 50% of all the vertebrate populations have been lost since 1970 [3–5].

Carnivores are among the animals that have experienced the most severe declines due to human activities such as habitat loss and fragmentation, illegal hunting and trade, conflict with livestock, and diseases transmitted by domestic animals [6–14]. Large carnivores are especially vulnerable to population losses because of their need for large areas, relatively small extant populations in comparison to medium and small carnivores, and slow population growth rates [15,16]. Globally, the population decline of many large cat species has been dramatic in recent times: tigers are only limited to 6% of its historic geographic range and their populations have declined 50% in the last decades. In a similar way lions occur only in 17% of their historic range and populations have declined 47% over the last 21 years [16–19]. Asiatic lions and cheetahs, for example, have disappeared from more than 98% of their historical range [13]; roughly 500 Asiatic lions live exclusively in the Gir forest in India and the population of Asiatic cheetahs in central Iran is less than 20 [15,20]. Other large cats, including leopards (*Panthera pardus*), snow leopards (*Panthera uncia*), and clouded leopards (*Neofelis nebulosa* and *N*. *diardi*) have experienced similar declines, and the leopard conservation status was recently uplisted by the IUCN to Vulnerable [13,21].

Large charismatic carnivores are good surrogates for biodiversity conservation [14], conservation priority policies, and conservation impacts at multiple scales [10]. Therefore, knowledge about carnivore current distribution and population trends is critical for conservation efforts. However, estimating population size of large cat species is challenging because of their large geographic and home ranges, elusiveness, and the fragmentation of the extant populations [13,17]. Early population estimates were based on field surveys carried out using transects and aerial counts [22]. In the last two decades, automatic camera traps have become widely used for assessing populations of many mammal species including large cats [23–26] and they have provided critical data for comprehensive large-scale censuses for species such as tigers, lions and jaguars [17,19,27,28].

The jaguar (*Panthera onca*) is the largest felid species in the Neotropics and the least studied in the genus *Panthera* [29]. Historically, jaguar geographic range extended from southwestern United States to central Argentina [27,30], but currently the species is considered Near Threatened globally because it has been extirpated from roughly 55% of its historic range and many of its populations are now either endangered or critically endangered [31–33]. Habitat destruction and fragmentation, retaliatory killing by cattle ranchers, illegal hunting, prey depletion, and introduced diseases, are the most significant threats to the long-term viability of jaguar populations [21,27].

Development and expansion of agricultural lands and livestock pastures, human settlements, roads and other infrastructure, are the primary causes of the loss and fragmentation of jaguar habitat [34–40]. In many communities, jaguars are perceived as a threat and killed by livestock ranchers in retaliation because the loss of livestock by predation is usually understood only from the economic loss perspective [41–46]. Additionally, jaguars are still hunted (albeit illegally) as trophies throughout their range, and recently illegal trade of their bones and teeth for the Chinese black-market has become an emerging threat [47]. The Convention on International Trade of Endangered Species (CITES) is taking action to curb this emerging threat [48].

Jaguars are still present across most of their historic range in Mexico. Its distribution extends along the lowlands and foothills of the mountains from Sonora to Chiapas in the Pacific coast, and from Tamaulipas to the Yucatan Peninsula in the Gulf of Mexico coast [49,50]. The main threats they face in the country are habitat loss and fragmentation, retaliatory killing, reduced abundance of their natural prey, and diseases transmitted by domestic animals [e.g., 50,51]. Recognizing these threats, the Mexican government prohibited jaguar trophy hunting in 1987 and listed it as an endangered species in 1994 [52].

In 2005, we organized a meeting of experts, which later became the National Alliance for Jaguar Conservation [53], to contribute to the long-term conservation of jaguars. We carried out basic ecological research, developed conservation and management initiatives, and helped to implement conservation policies. The Alliance developed a National Jaguar Conservation Strategy that identified nine fundamental and critical areas for jaguar conservation in Mexico: i) protection of biological corridors and identification of priority areas for conservation; ii) monitoring jaguar populations and its prey; iii) human-jaguar conflict resolution; iv) guidelines for translocation and reintroduction of jaguars; v) law and public policy; vi) mitigation of human infrastructure impact; vii) local community conservation; viii) environmental education; and ix) outreach [50,54–56]. They have become the framework for the Mexican government´s jaguar conservation program [50,56].

Periodic estimates of jaguar populations are essential to determine the vulnerability of the species and the structure and effectiveness of conservation policies and activities. We organized two national jaguar conservation censuses (*Cenjaguar*, the Spanish acronym) as a component of jaguar and its prey population monitoring program: the 2008–2010 period (hereafter, 2010 [54]) and the other 2016–2018 (hereafter, 2018). The 2010 national census [50,51,57] provided the baseline data against which other censuses, such as the recently completed 2018, can be compared. The main issues addressed in the present study are to: i) Estimate Mexico’s current jaguar population size; ii) evaluate its population trends by comparing the 2010 and 2018 census data; and iii) and identify the effectiveness of the National Jaguar Conservation Strategy and the long-term prospects of survival of the species in Mexico.

## Methods

### Records of occurrence

**Fig 1:**
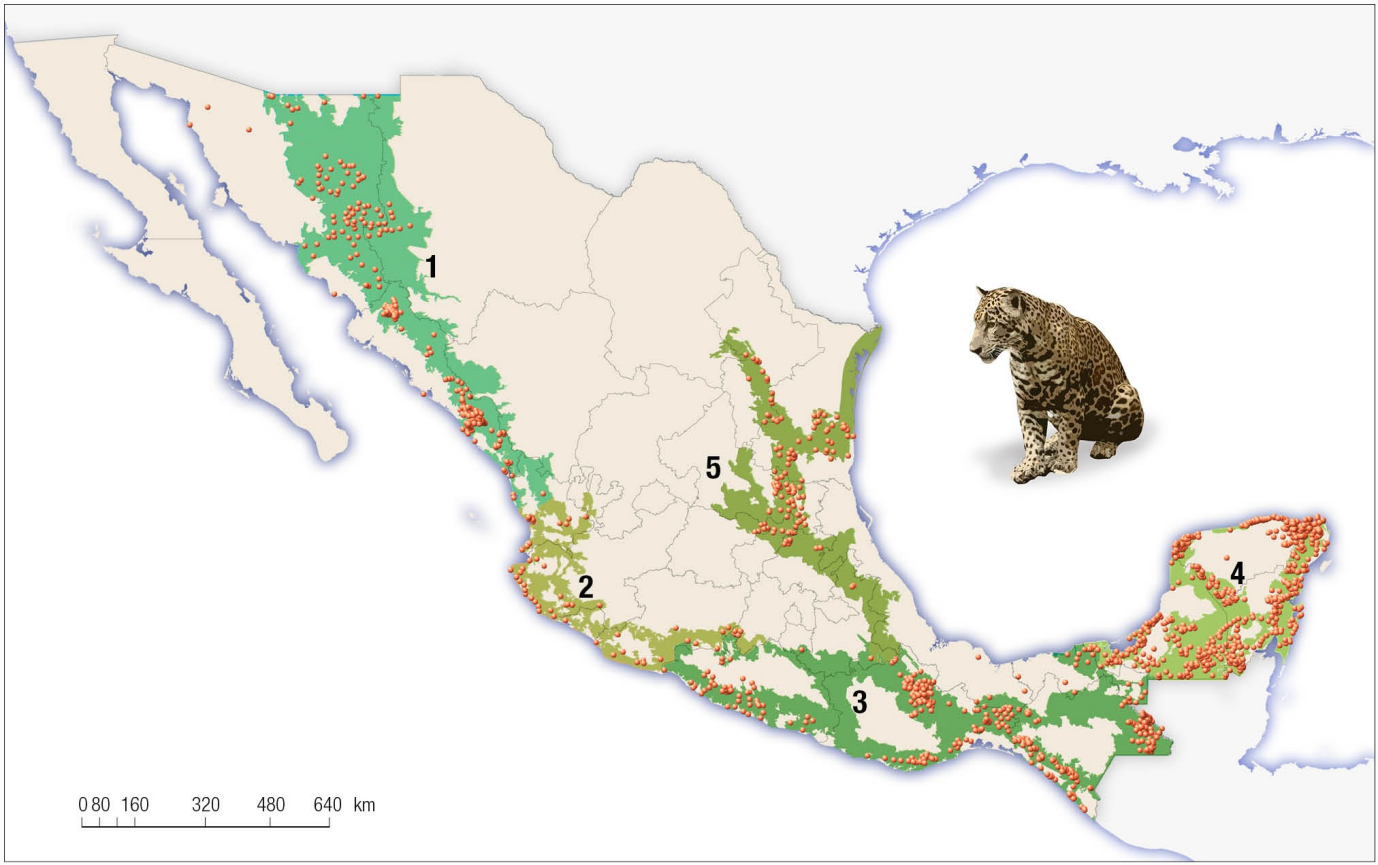
Current geographic range of jaguar (*Panthera onca*) in Mexico (green, Ceballos et al. 2018 [58]) and records in the last 20 years.

We developed a dataset of jaguar occurrences in Mexico over the past 20 years (1998–2018) from journal articles, technical reports, theses, books, other documentation, and unpublished records compiled by specialists from two workshops which included data from track signs, camera traps, GPS radio telemetry and jaguars killed. Most of the 15,632 records comprising the dataset came from Oaxaca, Chiapas, and the Yucatan Peninsula region in southern Mexico. Records in the country are from elevations ranging from sea level to about 3,000 masl (Fig 1).

### Camera trap surveys

**Fig 2:**
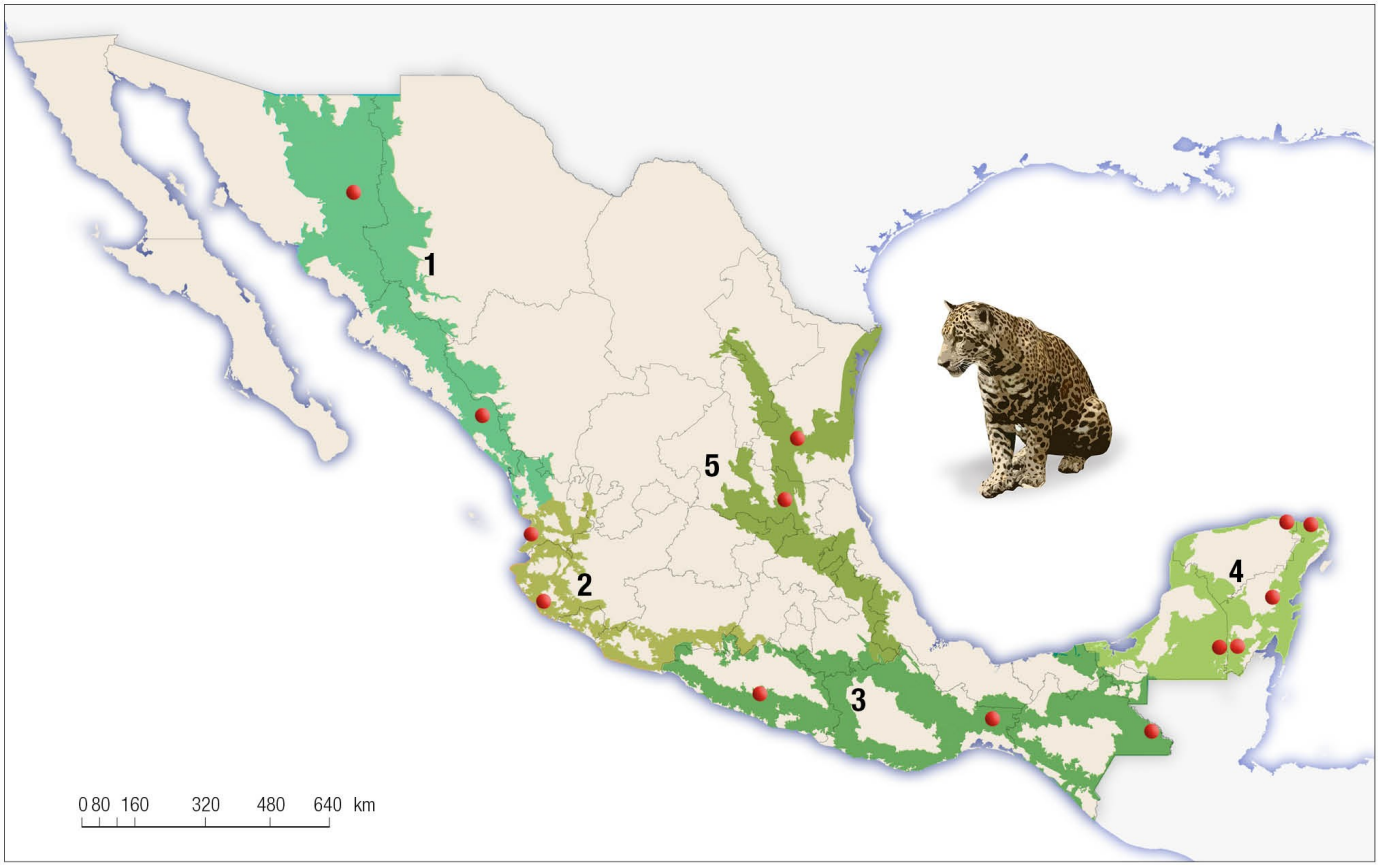
The five jaguar conservation regions include all the current geographic range of the species in Mexico and location study sites from the national jaguar census (CENJAGUAR).

Camera trap surveys were conducted at 13 sites in 2010 and 11 sites in 2018 (Table 1, Fig 2). Supplemental site information was gathered from multiple data sources [e.g., 29,54,57,59–63]. Under the Mexican environmental legislation no permit is required when studies are done with non-intrusive methods such as camera–traps (https://www.gob.mx/semarnat/acciones-y-programas/tramites-relacionados-al-tema-de-vida-silvestre). Sites were selected based on their importance as priority regions for jaguar conservation and knowledge that the working groups had of the areas. We used a standardized jaguar survey protocol designed for the first Cenjaguar [54,57,64], which facilitated comparison and analyses of similar datasets, and the surveys were carried out by personnel from multiple entities such as universities and non-governmental organizations.

**Table 1:** Summary data and statistics for the 2010 and 2018 jaguar censuses in Mexico.

**2010**								
**State**	**Sites**	**Effort**	**Sam Area**	**Photos**	**Captures**	**Recaptures**	**Climate**	**Habitat**
Sonora	Rosario	1020	126	3	2	2	Subtropical	Xeric scrublands and topical semideciduous forest
Sinaloa	San Ignacio	837	81	10	3	3	Warm Sub Humid	Tropical semideciduous and deciduous forests
Nayarit	Sierra de Vallejo	1035	81	12	4	3	Tropical Sub Humid	Tropical semideciduous and deciduous forests
Guerrero	Petatlán	2010	117	3	1	1	Subtropical	Tropical semideciduous and Pine forests
Oaxaca	Los Chimalapas	850	99	10	4	2	Tropical Humid	Tropical rainforest
Chiapas	Montes Azules	935	80	5	3	2	Tropical Humid	Tropical rainforest
Tamaulipas	Sierra de Tamaulipas	620	90	17	8	4	Subtropical	Tropical semideciduous forest
San Luis Potosí	San Nicolas de los Montes	837	99	10	3	3	Warm Sub Humid	Tropical semideciduous and Oak forests
Campeche	Calakmul	810	81	0	0	0	Tropical Sub Humid	Tropical semideciduous forest
Campeche	20 de Noviembre	810	81	1	1	1	Tropical Sub Humid	Tropical semideciduous forest
Quintana Roo	Caoba	800	117	22	5	3	Tropical Sub Humid	Tropical semideciduous forest
Quintana Roo	Noh Bec	552	90	15	11	3	Tropical Sub Humid	Tropical semideciduous forest
Quintana Roo	El Edén	802	81	45	6	3	Tropical Semi Humid	Tropical deciduous and semideciduous forests
**2018**								
**State**	**Sites**	**Effort**	**Sam Area**	**Photos**	**Captures**	**Recaptures**	**Climate**	**Habitat**
Sonora	Sahuaripa	1,080	271	3	2	1	Subtropical	Xeric scrubland and Topical semideciduous forest
Sinaloa	Cacaxtla	1,440	182	15	9	2	Warm Sub Humid	Tropical semideciduous and deciduous forests
Jalisco	Nevado de Colima	1,080	182	0	0	0	Tropical Sub Humid	Tropical semideciuos, Pine-Oak forests
Guerrero	Sierra de Chilpancingo	1,080	255	12	4	2	Subtropical	Tropical semideciduous and Pine Forests
Oaxaca	Los Chimalapas	1,080	156	10	3	3	Tropical Humid	Tropical rainforest
Chiapas	Montes Azules	1,608	359	12	4	6	Tropical Humid	Tropical rainforest
San Luis Potosí	Sierra del Abra Tanchipa	1,080	176	38	6	4	Tropical Sub Humid	Tropical deciduous forest
Campeche	Calakmul	1,080	186	28	9	6	Tropical Sub Humid	Tropical semideciduous forest
Quintana Roo	Laguna Om	1,440	248	25	6	3	Tropical Sub Humid	Tropical semideciduous and deciduous forests
Quintana Roo	El Edén	1,080	142	22	8	3	Tropical Semi Humid	Tropical deciduous and Semideciduous forests
Yucatán	Punto Put	1,080	405	29	5	3	Tropical Dry	Tropical deciduous forest

State: Indicate the state of Mexico, where the sampling sites were located. Sites: Indicate the name of the sampling sites. Simbology are: Effort: Number of trap-nights in the sample; SamArea: Effective sampling area (km^2^); Photos: Identifiable jaguar photos; Captures: Number of different jaguars identified; Recaptures: Number of jaguar photographed more than once. Climate: Indicates the type of climate prevailing in the sampling site; Habitat: Indicate the main vegetation type in the site.

The 2010 and 2018 national jaguar censuses were carried out in sampling locations located across the country and representing all vegetation types where the species is known to occur. Study sites (state, site, and plant association) for the *Cenjaguar 2010 (top)* are the following ones: 1) Sonora: Rosario (dry scrubland and subtropical deciduous forest); 2) Sinaloa: San Ignacio (tropical deciduous forest); 3) Nayarit: Sierra de Vallejo (tropical dry forest); 4) Guerrero: Petatlan (Tropical semideciduous and Pine forests); 5) Oaxaca: Los Chimalapas (tropical rain forest); 6) Chiapas: Montes Azules (tropical rain forest); 7) Tamaulipas: Sierra de Tamaulipas (tropical semideciduous forest); 8) San Luis Potosí: San Nicolas de los Montes (tropical semideciduous and Oak forests); 9) Campeche: Calakmul (tropical semigreen forest); 10) Campeche: 20 de Noviembre (tropical semideciduous forest); 11) Quintana Roo: Caoba (tropical semigreen forest); 12) Quintana Roo: Noh Bec (tropical semigreen forest), and; 13) Quintana Roo: El Edén (tropical deciduous forest).

The sites for the *Cenjaguar 2018 (bottom)* included the following ones: 1) Sonora: Sahuaripa (dry scrubland and subtropical deciduous forest); 2) Sinaloa: Meseta de Cacaxtla (tropical deciduous forest); 3) Colima: Nevado de Colima (tropical dry forest); 4) Guerrero: Sierra de Chilpancingo (pine-oak forest); 5) Oaxaca: Los Chimalapas (tropical rain forest); 6) Chiapas: Montes Azules (tropical rain forest); 7) Campeche: Calakmul (tropical semigreen forest); 8) Quintana Roo: Laguna Om (tropical semigreen forest); 9) Quintana Roo: El Edén (tropical deciduous forest); 10) Yucatan: Punto Put (tropical deciduous forest), and; 11) San Luis Potosí: Sierra del Abra-Tanchipa (tropical deciduous forest).

The 2010 survey was carried out from March 2008 to June 2010 and the 2018 survey from June 2016 to October 2018. In each study site, thirty-six cameras trap were set up across a ~81 km^2^ study area, in twenty-seven camera trap stations. The sampling cell was determined as the smallest conservatively estimated home range size for a female in the tropical forest (10 km^2^; 63, 64) in a given amount of time. The study area was divided into 9 sampling cells, each one of 9 km^2^, with two single and one double trap stations, Camera trap stations were strategically placed 1–2 km apart between each other to ensure complete coverage of the sampling area and where jaguar signs (e.g., tracks, scats, scrapes) were previously observed on the trails.

The 2010 survey was carried out for 30 days and the 2018 survey for 60 days. However, we used information for our analysis from only the first 30-day period of the 2018 survey to make the data collection period comparable to the 2010 survey. The mean effective sampling effort for each survey was ~800 camera trap days. We used Cuddeback® passive infra-red system (Non-Typical Inc., WI, USA) camera-traps Model E1 for the 2010 survey and Model E2 for the 2018 survey. The camera traps were placed 40–50 cm above the ground and at least 3 m off the trail where we expected jaguars to pass. The cameras remained active 24 hours per day and were configured to take pictures during 5 seconds with a 1-minute delay between photo events.

### Density estimates across the priority regions

We used a capture-recapture approach that requires identification of the individuals in the photos to estimate jaguar populations at each site. In order to minimize bias, experts reviewed all the photos of jaguars and made the identification based on their distinctive face and coat markings [65]. Each photo of an individual represented a capture. We compiled a capture history and generated a matrix where we assigned “1” to indicate the capture or recapture of a particular individual during the specific sampling episode and “0” to indicate that the individual was not recorded during the same sampling period [66].

To estimate abundance, we analyzed the capture-recapture history data using CAPTURE [24,66]. Our *a priori* hypothesis maintained that the heterogeneity of the capture model (*M*_*h*_) was a reasonable representation of the probability of jaguar capture, and the variability of the capture of individuals would be a better reflection of the demographic status of jaguars in comparison to other models available in CAPTURE [23,25,65,66].

In order to estimate jaguar density, we calculated the effective sampling area which included the area encompassed by the perimeter that was covered by the camera trap stations plus a buffer width outside this perimeter to include those individuals whose home ranges may be partially within the sampling area; we used the mean maximum distance moved (MMDM) to estimate this buffer width [64]. We followed Nichols & Karanth [67] to estimate jaguar densities for the 2018 survey in order to be consistent with the earlier 2010 Cenjaguar survey [54,57] and Karanth & Nichols [23] to calculate variances in density estimates. All estimates used from our study and complementary studies included the mean and lower and upper limits.

### Time-calibrated habitat suitability models

To assess jaguar population sizes for each of the two survey periods, we used time-calibrated habitat suitability models. This approach requires estimates of jaguar range at the specific time when the population surveys were conducted and pairs the occurrence data gathered over long periods with corresponding environmental conditions. Model outputs provide robust descriptions of how species select their habitat and reconstruction of habitat dynamics over the time [68–71].

We use the data compiled for the current jaguar distribution to generate the habitat suitability models and the Maxent version 3.4.1 [72] model to describe time-calibrated habitat suitability. This machine learning method generally outperforms other ecological niche model algorithms [73]. Maxent provides an estimate of the probability of distribution of a species based on the principle of maximum entropy and the assumption that all environmental constraints that regulate the species present are included in the model [74].

**Fig 3:**
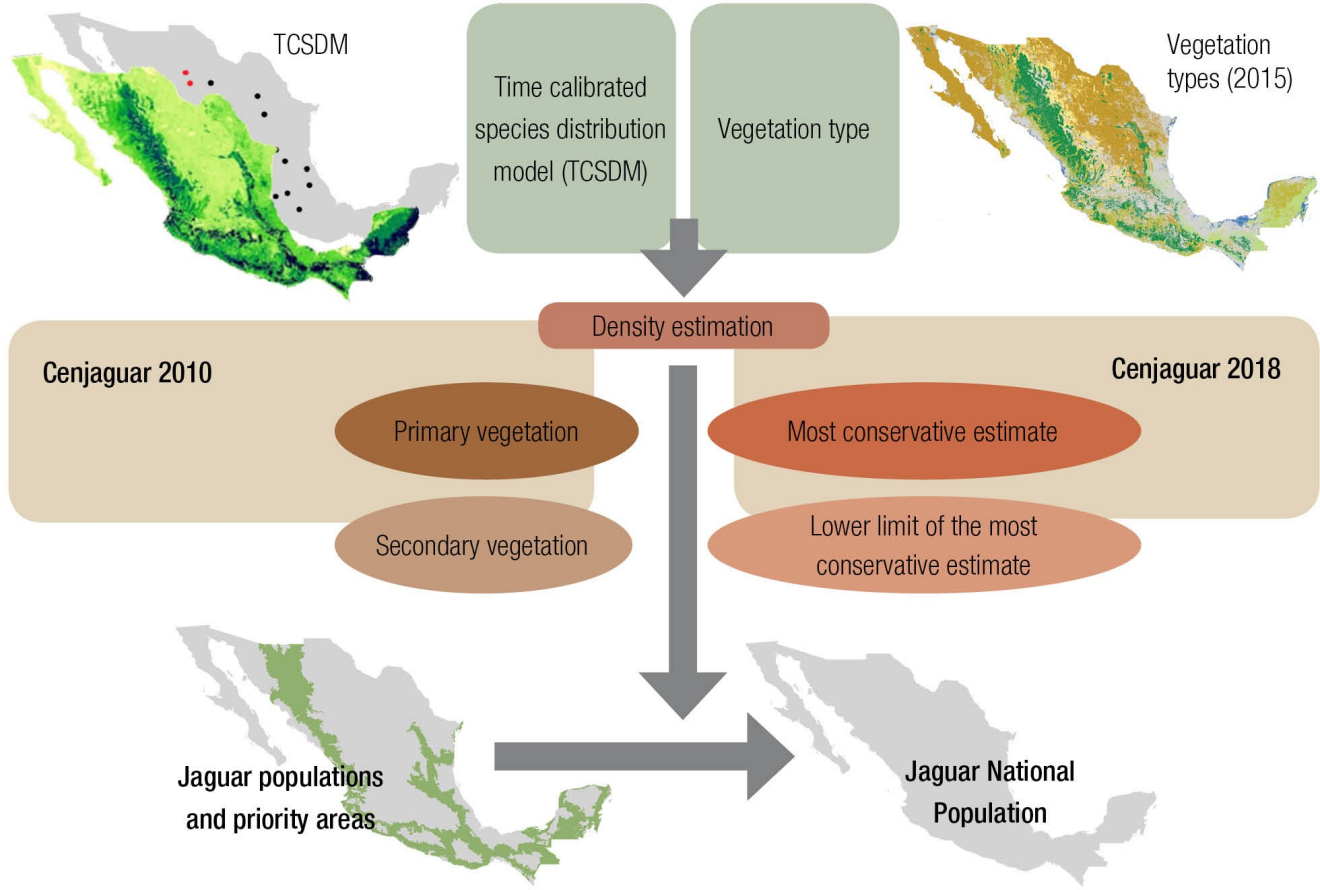
Diagram of the methodology used to estimate the jaguar population in Mexico. The methodology involves determining both the historic and current distribution of the species in Mexico (Top left) and the current natural vegetation cover of the country (Top right). Then using the density estimations in the field in 2010 and 2028, in primary and secondary vegetation with upper and lower estimate limits (middle). Finally, using those estimates and analyses to determine both the priority areas for conservation (lower left) and the national population estimates (lower right) for jaguars.

The methodology for our habitat suitability model (Fig 3) was similar to the approach used by Rodriguez-Soto et al., [49] and we assigned values ranging from 0 (unsuitable habitat) to 1 (optimally suitable habitat). To avoid model over-fitting, we used only quadratic and hinge features and a regularization multiplier of 1. We ran 10-fold cross-validation and assessed variable importance through jackknife estimations.

In order to reduce sampling bias of the 15,632 jaguar records compiled from Mexico, we applied a spatial filtering algorithm by randomly selecting occurrence records within a radius of 7.5 km, the radial distance of the average female home range size (180 km^2^) in southern Mexico and obtained a total of 622 records for the years 1998–2008 and 149 records for the years 2010–2018 for the analysis.

We sampled 50,000 random locations across all the country’s territory as background points and evaluated the predictive performance of our model by dividing the jaguar record locations randomly into two groups before model development: 80% of the data comprised a “model training” group and the remaining 20% comprised a “model testing” group for validation. We evaluated the performance of our model by calculating the area under the receiver operating characteristic curve (ROC).

Given that our models were generated to evaluate the areas across the country where jaguar presence would be more likely to occur for the years 2010 and 2018, we reclassified our original continuous models in two habitat suitability classes: low suitability (below the median suitability value of a probability of 0.55) and high suitability (above the median). We only considered the high suitability areas in all subsequent analysis because this model was used to extrapolate the densities of jaguars across the country.

For the time-calibrated habitat suitability models, we generated 1 km^2^ cell size raster layers for each of the three groups of predictor landscape variables and assumed these variables were important determinants of jaguar habitat (Table 2). One variable group was land cover and vegetation types. We used digital vegetation maps from the National Institute of Statistics and Geography Series IV and VI, for the years 2010 and 2018 respectively [75,76], and classified the land cover into eight classes: tropical rainforests, tropical dry forests, other forests, arid vegetation, grasslands, crops, seasonally flooded vegetation, and secondary vegetation. Data from the second group of landscape variables, elevation and slope, came from the Digital Elevation Model [77–80]. The third group of landscape variables was related to human infrastructure and includes density of towns (data for 2000 and 2010), distance to urban zones (data for 2009 and 2018), and distance to paved roads (data for 2008 and 2012; Table 3).

**Table 2:** Predictor variables used for modeling habitat suitability of jaguars in Mexico.

Type	Variable name	Source	Description	Expected effect	Justification
Land cover	Tropical rainforest	INEGI Serie VI	Areas covered by primary tropical forests	+	Jaguars are restricted mostly to areas of primary forest where they find their natural prey and refuge. Jaguars in Mexico occur in tropical rainforests, tropical dry forests, oaks and pine forests, and seasonally flooded habitats such as mangroves. Depict different vegetation types and their degradation degree by human activities for Mexico. This dataset also describes other land cover types such as crops, induced pastures, urbanized area and water.
	Tropical dry forest	INEGI Serie VI	Areas covered by primary dry forests	+	
	Other forest	INEGI Serie VI	Areas covered by primary oak, pine, cloud forests	+	
	Seasonally flooded vegetation	INEGI Serie VI	Areas covered by mangroves, and wetlands habitat types.	+	
	Arid vegetation	INEGI Serie VI	Areas covered by arid vegetation such as xeric scrubland vegetation types.	-	
	Secondary vegetation	INEGI Serie VI	Secondary vegetation of all the vegetation types.	+	Secondary vegetation class includes all vegetation types that present some kind of regenerative vegetation as an arboreal, shrub, and herbaceous.
	Crops	INEGI Serie VI	Areas of agriculture across the country	-	
	Grasslands	INEGI Serie VI	Areas associated to pastures for livestock.	-	
Terrain	Elevation	Digital Elevation Model	Elevation ranges across Mexico generated from a Digital Elevation Model (1 km) of all the country.	-	Jaguar habitat use is affected by different terrain conditions. Jaguars are frequently associated with lowland areas. Jaguar occupancy and movements would be hampered by the mountain ranges at higher altitudes].
	Slope	Digital Elevation Model	Slope values generated from a Digital Elevation Model (1 km) of all the country.	-	
Human perturbation	Distance to urban areas	INEGI	The minimum distance to the nearest urban area	+	Human activity affects habitat use by jaguars negatively due to disturbance and persecution. Information of urban areas, towns and roads was obtained from INEGI.
	Density of towns	INEGI	Density of towns around 7.5 km which is the radius of the female home range jaguars in southern Mexico	-	
	Distance to paved roads	INEGI	The minimum distance to the nearest paved roads	+	

**Table 3:** Variable contribution to the habitat suitability models for jaguars in Mexico, as used in Maxent.

Model	Variable	Percent contribution	Permutation importance	Observed effect
2010	Elevation	30.2	38.3	-
	Arid vegetation	22.1	5	-
	Tropical rainforests	9.2	7.3	+
	Secondary	8.4	15.4	+
	Crops	8.1	2	-
	Seasonally flooded vegetation	8	4.5	+
	Slope	2.8	4.5	+
	Tropical dry forest	2.6	4.8	+
	Distance to roads	2.3	5.8	+
	Other forests	2.2	7.6	+
	Density of towns	2.1	1.9	-
	Grasslands	1.2	1	+
	Distance to urban areas	0.9	2	+
2018	Arid vegetation	23.7	16.3	-
	Elevation	19.8	29.8	-
	Tropical rainforests	13.9	3.7	+
	Crops	13.5	14.4	-
	Grasslands	12	9	-
	Slope	6.2	7.9	+
	Distance to urban areas	4.6	6.3	+
	Density of towns	2.3	4.4	-
	Seasonally flooded vegetation	1.1	0.6	+
	Distance to roads	1.1	1.9	+
	Secondary vegetation	0.9	3.4	-
	Other forest	0.9	2.1	+
	Dry forest	0.2	0.3	+

### Sensitivity analysis

We ran sensitivity analysis to identify the optimal combination of thresholds for habitat suitability index. For each region, we generated a continuous positive and negative variation of 10% increases and decreases for each type of cover (primary and secondary) and for each region (given the differences in availability, ecological conditions, and density estimates). We then used the same extrapolation method for all regions in both years (2010 and 2018) and built a parametric sensitivity-analysis [81,82] for each year and each type of cover, relating the proportional change to the estimated population; we used as positive those values equal or larger than the median of the estimate. We identified the tendency and threshold of variation change, in terms of percentage changes in the different covers, regarding the significant change on the estimation. For this, we kept constant the baseline parameters and only the area ratio was altered at a time; these parameters of available vegetation area were altered in incremental and decremental (0.11) of the proportional area of each vegetation. Projections of estimated values were compared with those from our baseline estimation by plotting estimation values against the incremental model from the sensitivity analysis and estimating R^2^ [81,82]. If vegetation available for each region was highly sensitive larger changes were expected for the estimate and lower R^2^ values.

### Extrapolation of jaguar densities

To estimate the jaguar population size in Mexico, we first needed to determine the extent and location of suitable habitat. Prior studies have shown that jaguars in forested habitats prefer habitat with good vegetation cover and avoid areas modified by human activities such as agriculture, extensive pastures for cattle ranching, paved roads, towns, and so on [34–38]. We extracted all natural vegetation covers within both habitat suitability models, classified each cover according to primary and secondary vegetation types and estimated the area under each type. To estimate the areas, we used vegetation and land cover data layers at a scale of 1:250,000 from the National Institute of Statistics and Geography Series IV and VI [75,76]. Finally, we extrapolated the density estimates obtained by both Cenjaguar and other densities estimates for the country to the potential habitat for jaguars in Mexico.

**Fig 4:**
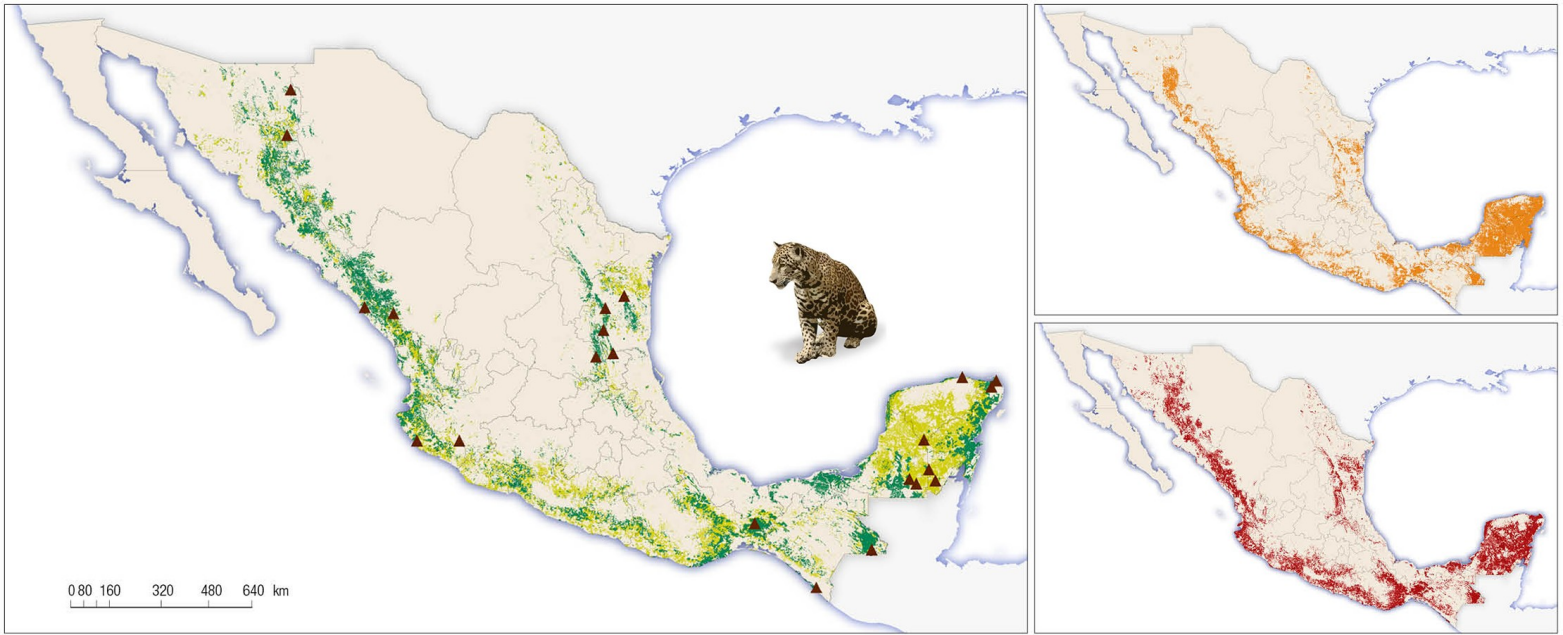
Vegetation types and their broad-scale distribution for 2018 and location study sites from CENJAGUAR within jaguar (a). Time-calibrated species distribution models for 2010 (b) and for 2018 (c) in Mexico.

**Fig 5:**
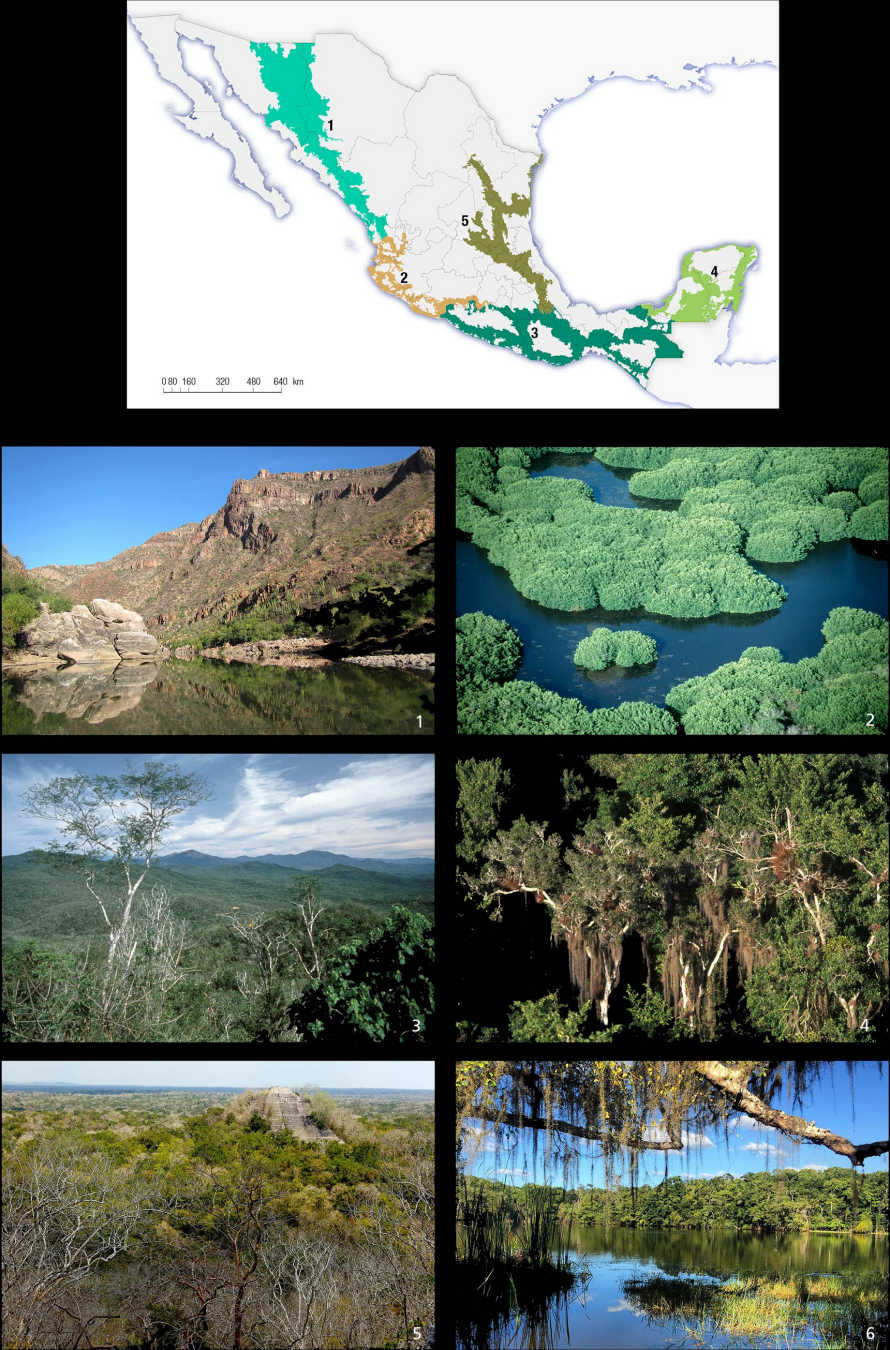
The five jaguar conservation regions and different vegetation types across current geographic range of the species in Mexico. The regions are the following ones: (1) Northern Pacific (Sonora and Sinaloa states); (2) Central Pacific coast (Nayarit, Jalisco, Colima, Michoacan, Mexico, and Morelos states); (3) South Pacific (Guerrero, Oaxaca, and Chiapas states); (4) Yucatan Peninsula (Yucatan, Campeche, and Quintana Roo states); and (5) Northeast and central Mexico (Tamaulipas, Nuevo Leon, San Luis Potosi, Queretaro, Puebla and Veracruz states. The vegetation types are as follows: (1) Scrubland (Nortthen Pacific region); (2) Mangrove (Central Pacific coast); (3) Tropical dry forest (Central Pacific coast region); (4) Temperate cloud forest (South Pacific region); (5) Tropical semegreen forest (Yucatan Peninsula); (6) Tropical rain forest (South Pacific region; All photos by Gerardo Ceballos).

The complete methodological process is depicted in Fig 3. In order to be as conservative as possible and to obtain weighted population numbers, we generated differential estimates for both primary and secondary vegetation (only with shrubby associations) types within the five conservations regions (Fig 4). Throughout the jaguar geographic range, we defined the following five ecogeographic regions for jaguar conservation in terms of the vegetation types and biogeographic affinities (Fig 5): 1. North Pacific coast (Sonora and Sinaloa); 2. Central Pacific coast (Nayarit, Jalisco, Colima, Michoacán, State of Mexico, and Morelos); 3. South Pacific coast (Guerrero, Oaxaca, Chiapas, and Tabasco); 4. Yucatán Peninsula (Campeche, Quintana Roo, and Yucatán); and 5. North central (Nuevo León, Tamaulipas, San Luis Potosí, Querétaro, Hidalgo, and Puebla).

We established each density estimate for every jaguar conservation region, and for each one, we used the lower density estimate (lowest values) to avoid any overestimation and weighted the number according to the available vegetation type (i.e., primary and secondary). For areas within each region classified as suitable habitat and primary vegetation, we used the lowest density estimate and extrapolated the potential number of individuals for the region; for those areas with suitable habitat but with secondary vegetation, we used the lower limit of the lowest density estimate and extrapolated the potential number of individuals for the region.

The techniques used in camera trapping can skew density estimates and most jaguar camera trap studies do not satisfy the requirements necessary to produce unbiased estimates and probably overestimate densities [83,84]. In our case, in order to avoid these biases, we conservatively used the lower limit of the lowest density estimate and applied the previously used Cenjaguar methodology. Nevertheless, our estimates of population size must be viewed with caution given that the information has a potential bias when extrapolating these results to different areas that incorporate the jaguar distribution range in Mexico.

## Results

### Distribution and habitat suitability models

Jaguars are still present in the tropical and subtropical regions of Mexico, from Sonora to Chiapas in the Pacific coast, and from Tamaulipas in the East coast to the Yucatan Peninsula in the south (Fig 1). Jaguars are more likely to be found in areas covered by tropical rain forest, tropical dry forest, seasonally flooded tropical forests, and mangroves. They are sometimes found in scrublands, clouds, pine and oak temperate forests adjacent to tropical regions. They are mostly absent in areas of arid vegetation, grasslands, and urban areas (Table 2). The areas identified by our models as with high suitability habitat include the coastal areas from Sonora (Pacific coast) and from Tamaulipas (Gulf coast) in the north of the country to the Yucatan Peninsula and Chiapas in the south, because they maintain large areas of well-preserved habitat. Furthermore, our model identified the Sierra Madre Oriental and Occidental, and the Balsas River basin as important areas for jaguars.

Our model for the 2010 survey identified ~267,063 km^2^ of high suitability habitat for jaguars in Mexico (roughly 14% of the country), yielding an Area Under the Curve (AUC) value of 0.85, whereas suitable habitat for the 2018 survey was ~288,890 km^2^ (14.8% of the country), yielding an AUC value of 0.87. These net values suggest a gain of ~20,000 km^2^ (8%) of suitable habitat for jaguars in the country between the two survey periods, that occurred primarily in the northern range of their distribution, in the states of Sonora and Sinaloa. In contrast, the models showed a loss of suitable habitat in the Yucatan Peninsula, one of the strongholds of jaguar conservation efforts in Mexico. According with our analysis between the 2010–2018 periods, jaguars have lost 19% and 4% of their range in tropical forest and flooded vegetation respectively, likely due to habitat transformation in southern Mexico. In the other hand, jaguars have gained range in dry forests (4%), arid vegetation (41%) and other forests (95%); this probably due to the increasing of jaguar knowledge in the northern region of Mexico in the last 10 years and the opportunistically adaptation of jaguars to exploit other suitable habitats.

### Density estimates across priority regions

**Fig 6:**
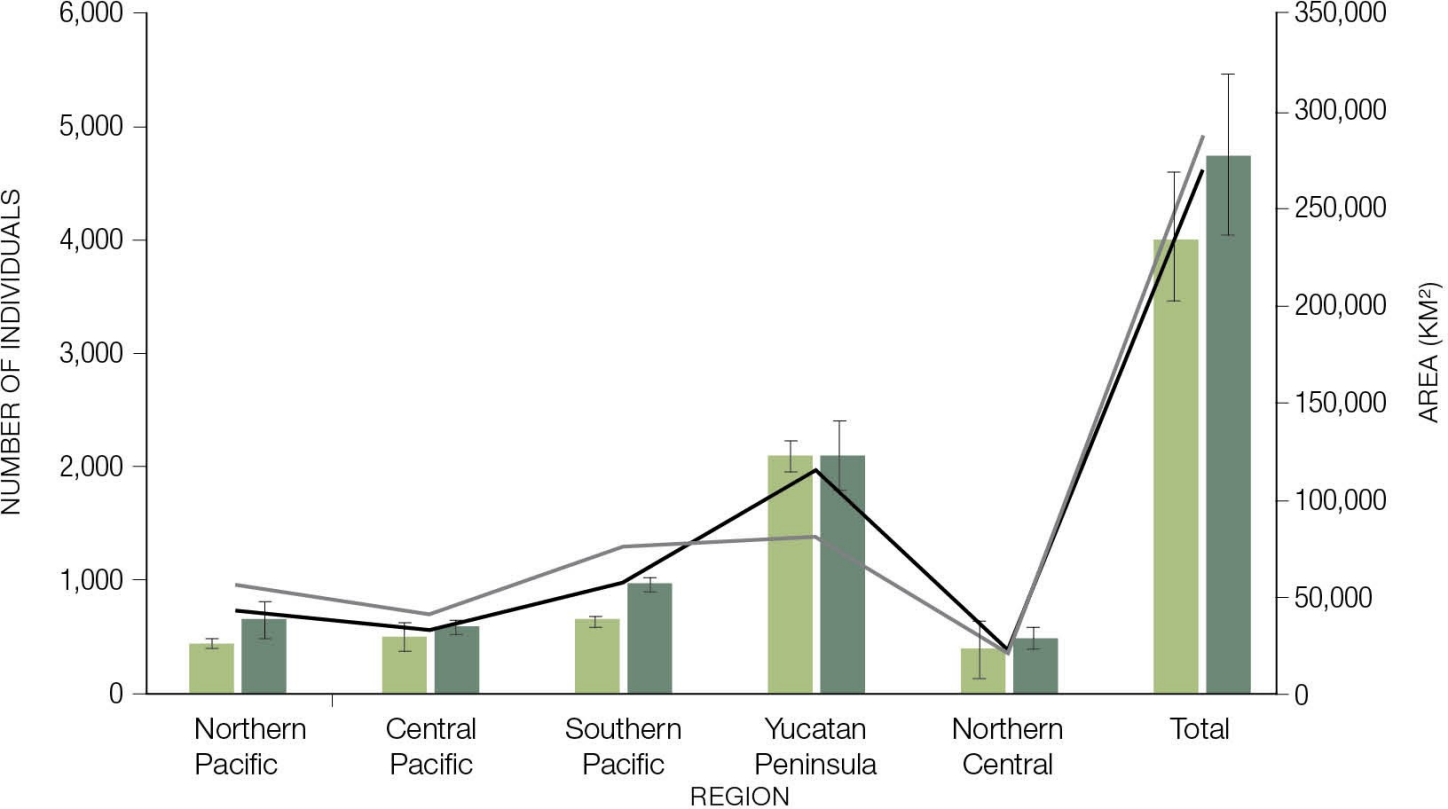
Jaguar population estimates in the 2010 (left bars) and for 2018 (right bars) censuses in Mexico. Suitable habitat available for 2010 (black line) and 2018 (gray line). Note: The population showed a ~20% increase in that period and suitable habitat also increased for four of the five regions and overall country.

Jaguar densities varied greatly across sites and slightly increased from 2010 to 2018 (Table 4, Fig 6). Average (±SD) jaguar density across all sampled sites was 2.8±1.2 ind/100 km^2^ for 2010 and 3.0±1.4 ind/100 km^2^ for 2018. Jaguar densities were lower in the drier subtropical sites in northwestern Mexico (e.g., 1.05 ind/100 km^2^ in Sonora) and higher towards the center of the country and especially in the Yucatan Peninsula region (e.g., 4.76 ind/100 km^2^ in El Edén).

**Table 4:** Estimates of potential jaguar populations for both censuses relative to vegetation type and available suitable area for each region.

Cenjaguar	Region	Vegetation type	Area in (km^2^)	Estimate	Total estimate
**2010**	1.North Pacific coast	Primary	26,606.4	298	437
		Secondary	13,931.8	139	
	2. Central Pacific coast	Primary	14,011.2	280	500
		Secondary	18,320.5	220	
	3. South Pacific coast	Primary	24,863.3	298	616
		Secondary	31,735.8	317	
	4.Yucatan Peninsula	Primary	33,456.7	887	2097
		Secondary	80,669.8	1210	
	5. North central	Primary	10,026.1	308	375
		Secondary	13,427.8	67	
	**TOTAL**			**4025±570**	
**2018**	1. North Pacific coast	Primary	41,488.5	506	644
		Secondary	16,784.7	138	
	2. Central Pacific coast	Primary	17,471.5	278	585
		Secondary	25,177.3	307	
	3. South Pacific coast	Primary	31,814.0	636	966
		Secondary	43,339.5	329	
	4.Yucatan Peninsula	Primary	28,251.8	913	2092
		Secondary	55,365.0	1179	
	5. North central	Primary	15,578.0	304	480
		Secondary	13,593.0	177	
	**TOTAL**			**4767±706**	

### Sensitive analysis

**Fig 7:**
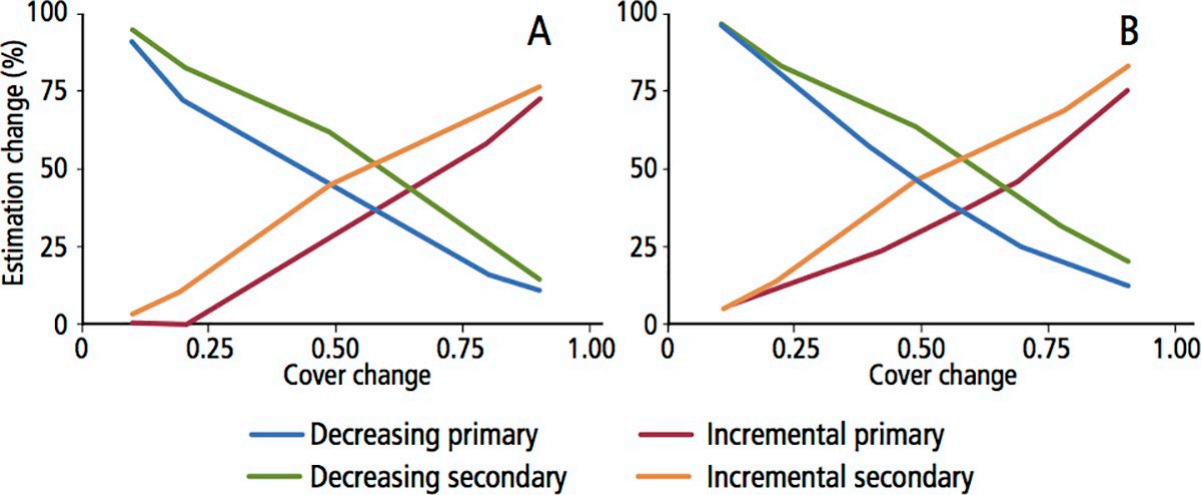
Sensitivity analysis of jaguar estimations in relation to changes in primary and secondary vegetation in the (A) 2010 and (B) 2018 censuses in Mexico. The thresholds for significant changes in our estimations are above 0.50 in all scenarios. This means that our jaguar population density estimations would change only if the vegetation changes would be over 50%, indicating the robustness of our the thresholds for significant changes in our estimations are above 0.50 in all scenarios analyses.

In terms of model sensitivity, time-calibrated habitat suitability models showed that variables such as elevation, arid vegetation and tropical forests showed the most significant contribution to the overall performance of the model, accounting for 61.5% of the total contribution for 2010, and 57.4% for the 2018 model (Table 3). For both vegetation types in both years, estimates have apparent low sensitivity to changes in the amount of cover (Fig 7); in all cases, sensitivity curves indicate that the thresholds for significant changes in our estimations are above 0.50 in all scenarios. Furthermore, for both cover types in 2018 and secondary vegetation in 2010, R^2^ values were low (0.05, 0.05 and 0.11, respectively), while primary vegetation in 2010 showed a larger R^2^ value (0.11) and relatively more significant change.

### Jaguar population size 2010–2018

Interestingly, there was a 20% increase on the average jaguar population estimate in Mexico between 2010 and 2018. Based on the analysis of the suitable habitat and extrapolations from jaguar abundance estimates in each survey (2010, 2018) in the five priority regions, we calculated a total jaguar population in Mexico of 4,025 (±570) in 2010 and 4,766 (±706) in 2018. The largest estimated numbers of individuals were located in the Yucatan Peninsula in both surveys, with an estimate of over 2,000 jaguars for the two censuses. The lowest value was in the northern central region, with estimates of 375 (±233) in 2010 and ~480 (±101) in 2018 (Table 3). Most regions across the country showed a significant increase in the number of potential individuals (Table 3), and the largest variation in the number of estimated individuals occurred in the southern Pacific region (Fig 6).

## Discussion

This study is the first nation-wide field data-based assessment of jaguar abundance and population size in a country throughout its distribution range. It represents an important step forward in the conservation efforts for this species because it establishes a baseline database and highlights the need and value of large-scale, national population assessments. Similar efforts for jaguars and other large cats, such as tigers and lions, have been extremely useful in the guidance of conservation needs and actions [1,24,78,83,85–88].

Our data shows that the jaguar population in Mexico has increased in the past 8–10 years. This time-period framework represents the evaluation of jaguar conservation status in approximately one jaguar generation length (~ 6.8 years) [32]. The censuses provided critical data that advanced our understanding of the life history and needs of jaguars and critical guidance for effective conservation measures. Other studies, based on continent-scale models, have estimated similar population numbers for Mexico. For example, using the same regional populations as we did, de la Torre et al. [33] estimated a total population of 2,860 jaguars, whereas a study by Jedrzejewski et al. [89], which used a coarser data set and one that was not specific to Mexico, estimated a population of 4,343 (3,400±5,383), which approximated to our calculated number.

Based on the results from the 2010 survey, the National Alliance for Jaguar Conservation in Mexico (ANCJ; Spanish acronym) defined priority conservation actions to reduce the most pressing threats to jaguar populations across the country, primarily habitat fragmentation, road and other infrastructure development, human-wildlife conflicts, inappropriate tourism development, and poaching [49,50]. While these threats are common throughout the jaguar range [33], specific and targeted conservation actions in Mexico were designed for each region under the ANCJ umbrella.

The 2010–2018 increases despite human population growth, expansion of infrastructure, illegal hunting, and other threats, may be explained by a combination of factors. For example, there has been an increment in tropical land cover with suitable jaguar habitat, especially in western Mexico; protection of nature reserves such as biosphere reserves; payment of environmental services to landowners located on jaguar habitat; law enforcement; community-based conservation and education; and cattle insurance for jaguar predation [39,49,50,90,91]. It is interesting to note that the Yucatan Peninsula region, which has the largest jaguar population in Mexico, showed large habitat loss and, as expected, little increase in the population.

Collective and coordinated actions at regional and national scales are required to ensure sustainable and healthy populations in Mexico. Our results highlight the importance of integrating solid, coordinated science initiatives to inform decision-making for successful conservation [82,92]. More limited, isolated, research and conservation approaches are useful to focus at regional scales, but are insufficient to develop a national conservation strategy. Such large-scale strategy is fundamental to try maintaining jaguars in Mexico and any other country.

The Cenjaguar has identified areas throughout the country which have varying degrees of isolation and other attributes important for jaguar conservation, the spatial distribution of their populations, and estimates of their potential sizes, information that is essential to effective conservation pathways [49–51,81,93,94]. Further refinements and analyses of these and other potential habitat areas [49] will provide additional useful and realistic evaluations of their conservation potential.

Our study shows that focusing on strategic components tackling the most urgent threats for jaguar survival is fundamental for maintaining the populations. We believe our Cenjaguar approach, which is collaborative, inclusive, systematic, and at the national scale, offers the best opportunity to provide high-quality scientific information for jaguar conservation in Mexico [50,90]. The conservation of such iconic species in Mexico requires further reduction of the main human-related threats common along the jaguar’s geographic range, such as habitat loss and hunting [27] and the protection of all the remaining the habitat identified as critical for the survival of the species.

The permanence of viable jaguar populations in Mexico will crystalize the vision and efforts of many people involved in jaguar conservation past and present. It will show that the coexistence of jaguars and people is possible if appropriate measures are taken. And it will offer hope for Mexico, its biological diversity, and its people in the most challenging and dangerous time that has been faced by humanity.

## Supporting information

S1 File (XLSX)
