# Correction: Beyond words: From jaguar population trends to conservation and public policy in Mexico

**DOI:** 10.1371/journal.pone.0305642

**Published:** 2024-06-12

**Authors:** Gerardo Ceballos, Heliot Zarza, José F. González-Maya, J. Antonio de la Torre, Andrés Arias-Alzate, Carlos Alcerreca, Horacio V. Barcenas, Gerardo Carreón-Arroyo, Cuauhtémoc Chávez, Carlos Cruz, Daniela Medellín, Andres García, Marco Antonio Huerta-García, Marco A. Lazcano-Barrero, Rodrigo A. Medellín, Oscar Moctezuma-Orozco, Fernando Ruiz, Yamel Rubio, Victor H. Luja, Erik Joaquín Torres-Romero

The 13^th^ author’s name is spelled incorrectly. The correct name is: Marco Antonio Huerta-García.
