# Correction: Beyond words: From jaguar population trends to conservation and public policy in Mexico

**DOI:** 10.1371/journal.pone.0326314

**Published:** 2025-06-16

**Authors:** Gerardo Ceballos, Heliot Zarza, José F. González-Maya, J. Antonio de la Torre, Andrés Arias-Alzate, Carlos Alcerreca, Horacio V. Barcenas, Gerardo Carreón-Arroyo, Cuauhtémoc Chávez, Carlos Cruz, Daniela Medellín, Andres García, Marco Antonio Huerta-García, Marco A. Lazcano-Barrero, Rodrigo A. Medellín, Oscar Moctezuma-Orozco, Fernando Ruiz, Yamel Rubio, Victor H. Luja, Erik Joaquín Torres-Romero

After publication of this article [1,2], concerns were raised by a reader about the methodology and the data availability. After comprehensive correspondence with the authors and assessment by a member of the Editorial Board, the article’s overall results and conclusions are upheld, but some areas that needed clarification were identified

The authors have provided the following corrected tables, figures and supplemental data files.

There were some errors in Table 1. Please see the correct Table 1 here and the complete, correct Table 1 caption here.

**Table 1:** Summary data and statistics for the 2010 and 2018 jaguar population estimates in Mexico.

2010								
**State**	**Sites**	**Effort**	**Sam** **Area**	**Photos**	**Captures**	**Recaptures**	**Climate**	**Habitat**
Sonora	Rosario	1020	126	6	2	2	Subtropical	Xeric scrublands and topical semideciduous forest
Sinaloa	San Ignacio	837	81	42	6	6	Warm Sub Humid	Tropical semideciduous and deciduous forests
Nayarit	Sierra de Vallejo	1035	81	24	4	3	Tropical Sub Humid	Tropical semideciduous and deciduous forests
Guerrero	Petatlán	2010	117	3	1	0	Subtropical	Tropical semideciduous and Pine forests
Oaxaca	Los Chimalapas	850	99	10	4	2	Tropical Humid	Tropical rainforest
Chiapas	Montes Azules	935	80	5	3	2	Tropical Humid	Tropical rainforest
Tamaulipas	Sierra de Tamaulipas	620	90	17	8	4	Subtropical	Tropical semideciduous forest
San Luis Potosí	San Nicolas de los Montes	837	99	10	3	2	Warm Sub Humid	Tropical semideciduous and Oak forests
Campeche	Calakmul	810	81	0	0	0	Tropical Sub Humid	Tropical semideciduous forest
Campeche	20 de Noviembre	810	81	1	1	0	Tropical Sub Humid	Tropical semideciduous forest
Quintana Roo	Caoba	800	117	22	5	3	Tropical Sub Humid	Tropical semideciduous forest
Quintana Roo	Noh Bec	552	90	15	11	3	Tropical Sub Humid	Tropical semideciduous forest
Quintana Roo	El Edén	802	81	45	6	3	Tropical Semi Humid	Tropical deciduous and semideciduous forests
**2018**								
**State**	**Sites**	**Effort**	**Sam Area**	**Photos**	**Captures**	**Recaptures**	**Climate**	**Habitat**
								
Sonora	Sahuaripa	1,080	271	3	2	1	Subtropical	Xeric scrubland and Topical semideciduous forest
Sinaloa	Cacaxtla	1,440	182	15	9	2	Warm Sub Humid	Tropical semideciduous and deciduous forests
Jalisco	Nevado de Colima	1,080	182	0	0	0	Tropical Sub Humid	Tropical semideciuos, Pine-Oak forests
Guerrero	Sierra de Chilpancingo	1,080	255	12	3	2	Subtropical	Tropical semideciduous and Pine Forests
Oaxaca	Los Chimalapas	1,080	156	10	3	2	Tropical Humid	Tropical rainforest
Chiapas	Montes Azules	1,608	359	12	5	1	Tropical Humid	Tropical rainforest
San Luis Potosí	Sierra del Abra Tanchipa	1,080	176	38	6	4	Tropical Sub Humid	Tropical deciduous forest
Campeche	Calakmul	1,080	186	28	7	1	Tropical Sub Humid	Tropical semideciduous forest
Quintana Roo	Laguna Om	1,440	248	25	6	2	Tropical Sub Humid	Tropical semideciduous and deciduous forests
Quintana Roo	El Edén	1,080	142	22	11	8	Tropical Semi Humid	Tropical deciduous and Semideciduous forests
Yucatán	Punto Put	1,080	405	29	3	1	Tropical Dry	Tropical deciduous forest

Symbology, State: Mexican state where the site was located; Sites: indicate the name of study site; Effort: trap-nights; SamArea: Effective sampling area (km^2^); Photos: total number of jaguar photos including non-independent records (i.e., more photos than the sum of captures and recaptures); Captures: number of different jaguars identified in the camera trap – data; Recaptures: number of jaguars recorded more than once. Climate: indicates the type of climate prevailing in the sampling site; Habitat: indicate the main vegetation type in the site.

The data points for the surveyed localities in 2010 and 2018 in Fig 4 are incorrect. Please see the correct Fig 4 and the complete, correct Fig 4 caption here.

**Fig 4:**
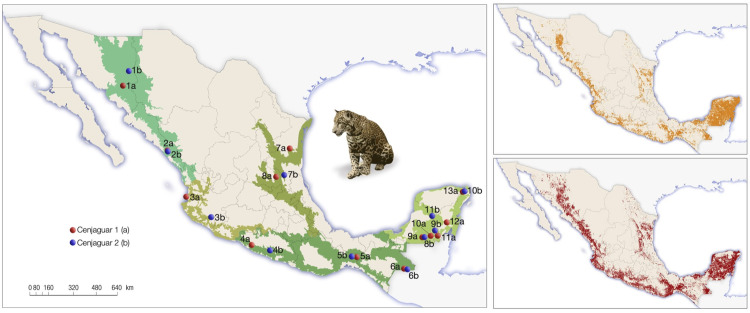
Vegetation types and their broad-scale distribution for 2018 and location study sites from CENJAGUAR (left) within State (Sites): 2010: 1a: Sonora (Rosario); 2a: Sinaloa (San Ignacio); 3a: Nayarit (Sierra de Vallejo); 4a: Guerrero (Petatlán); 5a: Oaxaca (Los Chimalapas); 6a: Chiapas (Montes Azules); 7a: Tamaulipas (Sierra de Tamaulipas); 8a: San Luis Potosí (San Nicolas de los Montes); 9a: Campeche (Calakmul); 10a: Campeche (20 de Noviembre); 11a: Quintana Roo (Caobas); 12a: Quintana Roo (Noh Bec); 13a: Quintana Roo (El Edén). 2018: 1b: Sonora (Sahuaripa); 2b: Sinaloa (Cacaxtla); 3b: Jalisco (Nevado de Colima); 4b Guerrero (Sierra de Chilpancingo); 5b: Oaxaca (Los Chimalapas); 6b Chiapas (Montes Azules); 7b: San Luis Potosí (Sierra del Abra Tanchipa); 8b Campeche (Calakmul); 9b: Quintana Roo (Laguna Om); 10b: Quintana Roo (El Edén); 11b Yucatán (Punto Put). Time-calibrated species distribution models for 2010 (Upper right) and for 2018 (Lower right) in Mexico.

The Data Availability statement for this article is incorrect. The authors have shared the geographical coordinates of the study sites for editorial assessment, but as per PLOS’ data sharing policy, location data on endangered species is considered sensitive data and we will not make this dataset publicly available. The authors confirm that all data underlying the findings are fully available without restriction except the geographical coordinates of the study sites. This dataset is available upon request from the Data Governance Committee from the Alianza Nacional para la Conservación del Jaguar A.C. at aibioconservacion@correo.ler.uam.mx.

There were missing points in the S1 File. Please see the complete, correct dataset here.

In addition, the authors provide additional information to clarify the following areas of expressed concern.

## 1. The calculation of the density estimates

The density estimates were calculated as follows:

The authors developed field surveys in 24 sites and used available estimations for both periods (2010 and 2018) based on a 27 camera-trap stations array for 30 days (2010 survey) and 60 days (2018 survey). The surveys covered a total area of ca.81 km2, with a mean distance of 1.5 km. Sites were selected according to their importance as priority regions for jaguar conservation [3,4] and previous knowledge of the working groups.

The authors identified jaguar individuals by data obtained by camera trap surveys and used recapture models to estimate densities. Capture-recapture models were estimated after building capture histories for each site with 30-days sampling periods; we then estimated abundance values under seven models that differ in the source of variation in capture probability (i.e., individual heterogeneity, behavior, time, and combinations). The best model was selected using a discriminant function developed by [5] and canonical estimators developed by [6]. Camera locations were used to develop a Minimum Convex Polygon (MCP) between stations, to which we added a buffer based on the Mean Maximum Distance Moved (MMDM) and estimated the density as the abundance over the MCP and buffer area. Density estimates were obtained for different representative habitat types for jaguar landscapes in Mexico. In 2005, the Commission for Protected Natural Areas (CONANP), SEMARNAT, and the National Autonomous University of Mexico held the 1st Symposium on the Mexican Jaguar in the 21st Century [3]. More than 25 experts on the jaguar in Mexico participated in this event, and among the topics discussed were the priority areas for jaguar conservation in Mexico. This exercise of prioritization of areas was based on the following criteria: i) presence of jaguar populations, recent studies or expert knowledge; ii) presence of suitable habitat within the current distribution area of the species, based on recent records of jaguars and expert knowledge; and iii) presence of isolated records of jaguars. Based on these criteria, the prioritization generated three groups: Priority I, those regions that still maintain jaguar populations; Priority II, regions that have considerable extensions of suitable habitat for the jaguar, but the presence of the species has not been systematically evaluated; and Priority III regions where there are isolated records of jaguars, supported by systematic studies or anecdotal observations, but which no longer have considerable areas of natural vegetation that would allow for the persistence of a jaguar population. Experts agreed upon these regions [7], taken up in various Mexican government documents [8], and supported by recent publications [4,9–11].

Potential distribution models were implemented using jaguar records and landscape information for both periods of time (2008 and 2018). The authors built a time-calibrated species distribution model using available information for both times to properly identify the available habitat coverage in each of the previously identified jaguar conservation regions. Details on each of the models are provided in the Methods section. The authors estimated habitat suitability for jaguars for both periods using the time-calibrated species distribution models, discriminating between primary and secondary vegetation.

Based on the density estimation, the authors calculated 95% confidence intervals for each site estimation and for each region to have the most conservative estimates based on the lower intervals for each region. The authors then had mean, maximum, and minimum density estimates on each region based on multiple estimations. Using the most conservative estimate for available primary vegetation habitat and the lower limit (lower interval) of the most conservative estimate for secondary vegetation habitats, the authors extrapolated the number of individuals to the total available habitat within suitable environmental conditions calibrated for each time-period for the region. Finally, with total estimations per region, the authors estimated Mexico’s most conservative number of individuals, calibrated by time-period and vegetation types (optimal and suboptimal habitats).

## 2. The use of CAPTURE model for estimating densities

The use of the MMDM, based on closed population capture-recapture approaches, estimates were positively correlated with the density estimates, while the use of the Mh, MMDM and SECR models produced very similar results [12]. The authors used the mean maximum distance moved (MMDM) to estimate buffer width under a capture-recapture framework. Although they acknowledge that SECR is a more recent approach, the authors consider that classic capture-recapture models are still a valid approach, as reflected in the literature [13,14]. The authors acknowledge the limitations in interpreting their results because the density estimates are different across habitats, regions, and periods and, that local and regional scale population monitoring programs are still necessary to better understand the estimation in remote regions. However, the authors understand that CAPTURE is now not considered the most reliable approach, this was the best model, considering the data limitations, when they did their analysis.

## 3. The absence of jaguar contacts in areas where jaguar is known to be present

It was unexpected that there were no records in Calakmul in the 2018 sampling period as this study site is known to have good number of jaguars [15,16]. The authors report that camera trap malfunction was discarded as the camera traps recorded other mammals in the area. The authors suggest a logging prospection study undergoing in the area as a possible cause for the absence, as jaguars leave the sampling and other sites when unusual human activities occur [17–19].

## Supporting information


S1 File.
(XLSX)
